# Gedunin Mitigates *Cutibacterium acnes*-Induced Skin Inflammation by Inhibiting the NF-κB Pathway

**DOI:** 10.3390/ph18010071

**Published:** 2025-01-09

**Authors:** Ju Kyoung Sim, Ye Ji Heo, Jin Hak Shin, Seon Sook Kim, Su Ryeon Seo

**Affiliations:** 1 Department of Molecular Bioscience, College of Biomedical Science, Kangwon National University, Chuncheon 24341, Republic of Korea; tla0156@kangwon.ac.kr (J.K.S.); hyj001001@kangwon.ac.kr (Y.J.H.); wlsgkr1876@kangwon.ac.kr (J.H.S.); 2 Institute of Life Science, Kangwon National University, Chuncheon 24341, Republic of Korea; painniche@kangwon.ac.kr; 3 Institute of Bioscience and Biotechnology, Kangwon National University, Chuncheon 24341, Republic of Korea

**Keywords:** *Cutibacterium acnes* (*C. acnes*), gedunin, skin inflammation, NF-κB, NLRP3 inflammasome, acne vulgaris

## Abstract

**Background/Objectives**: *Cutibacterium acnes (C. acnes)*, a bacterium residing in hair follicles, triggers acne by inducing monocyte-mediated inflammatory cytokine production. Gedunin, a limonoid derived from *Azadirachta indica* (commonly known as neem), is renowned for its antifungal, antimalarial, anticancer, anti-inflammatory, and neuroprotective effects. However, its role in mitigating *C. acnes*-induced skin inflammation remains unexplored. This study investigates the anti-inflammatory effects of gedunin on *C. acnes*-induced skin inflammation and elucidates the underlying mechanisms. **Methods**: The anti-inflammatory activity of gedunin was assessed using RAW 264.7 mouse macrophage cells and mouse bone-marrow-derived macrophages (BMDMs). Key inflammatory mediators, including interleukin-1β (IL-1β), tumor necrosis factor-α (TNF-α), inducible nitric oxide synthase (iNOS), cyclooxygenase-2 (COX-2), and interleukin-6 (IL-6), were evaluated. Mechanistic studies focused on the nuclear factor-kappa B (NF-κB) and mitogen-activated protein kinase (MAPK) signaling pathways, along with the NOD-like receptor pyrin domain-containing 3 (NLRP3) inflammasome. An in vivo acne model was employed to examine gedunin’s therapeutic efficacy. **Results**: Gedunin significantly reduced the expression of IL-1β, TNF-α, iNOS, COX-2, and IL-6 in RAW 264.7 cells. It inhibited NF-κB activation without affecting the MAPK pathways, including JNK/SAPK, ERK, and p38 MAPK. Gedunin also suppressed the activation of the NLRP3 inflammasome in BMDMs. In the mouse acne model, gedunin effectively alleviated *C. acnes*-induced inflammation, primarily by targeting NF-κB signaling. **Conclusions**: Gedunin demonstrates potential as a therapeutic agent for acne treatment by targeting key inflammatory pathways, particularly NF-κB signaling. This study highlights gedunin’s promise as an alternative approach to managing *C. acnes*-induced skin inflammation.

## 1. Introduction

Acne is a persistent inflammatory condition of the pilosebaceous unit, affecting approximately 70–80% of teenagers and young adults [1,2]. The pilosebaceous unit consists of hair, follicles, and sebaceous glands. Within these units, three coexisting microbial groups are present: lipophilic yeasts (*Pityrosporum* species), anaerobic diphtheroids (*Cutibacterium acnes* and *Propionibacterium granulosum*), and Gram-positive coagulase-negative cocci (staphylococci and micrococci) [3]. Among these, *C. acnes* is known to play a significant role in triggering the inflammatory response in acne [1]. *C. acnes* is a Gram-positive anaerobic bacterium that proliferates excessively within the follicle and acts as an immune stimulant by secreting various pro-inflammatory cytokines that are crucial for the manifestation of skin inflammation [4]. The role of *C. acnes*, which resides in the pilosebaceous unit, has been demonstrated by multiple studies. Many patients with acne have shown increased cellular and humoral immunity against *C. acnes* [5,6].

Microbial agents and their derivatives stimulate keratinocytes to produce pro-inflammatory cytokines, chemokines, and antimicrobial peptides through signaling pathways involving toll-like receptors (TLRs). Research highlights that *C. acnes* stimulates macrophages to release IL-6, but this response is contingent on TLR2 [7]. Macrophages from wild-type mice, as well as those lacking TLR6 or TLR1, successfully secrete IL-6 in response to *C. acnes*. In contrast, TLR2-deficient macrophages fail to produce IL-6, underscoring the critical role of TLR2 in mediating this immune response. Lipopolysaccharide (LPS), a commonly used inflammatory agent, is part of the cell wall of Gram-negative bacteria and is recognized by TLR4, setting it apart from the signaling pathway triggered by *C. acnes*. Upon the recognition of pathogen-associated molecular patterns (PAMPs) and damage-associated molecular patterns (DAMPs) from *C. acnes* by TLR2, the NF-κB and MAPK pathways become activated, which leads to the expression of inflammation-related genes, including TNF-α, IL-1β, and IL-6 [8,9].

NF-κB controls the expression of multiple molecules, including pro-inflammatory cytokines, chemokines, adhesion molecules, and inducible enzymes, making it essential for both innate and adaptive immunity. Notably, the NF-κB-mediated upregulation of COX-2 and iNOS enhances the intracellular production of nitric oxide (NO) and prostaglandin E2 (PGE2) [10,11]. NF-κB also functions as a transcription factor that drives the expression of pro-IL-1β and NLRP3, playing a pivotal role in regulating the inflammasome signaling pathway. Cellular stress or danger signals, including ATP release, reactive oxygen species (ROS), or ion flux disturbances, trigger the assembly of the inflammasome complex. The NRLP3 inflammasome recruits the adaptor protein apoptosis-associated speck-like protein containing a CARD (ASC), which interacts with caspase-1, forming a multi-protein complex. Caspase-1 is then activated and cleaves pro-IL-1β into its mature form, IL-1β [12]. The mature IL-1β is subsequently secreted, driving the inflammation and immune responses. The dysregulation of this process can lead to uncontrolled inflammation and is implicated in various inflammatory diseases.

*Azadirachta Indica* (commonly known as neem) belongs to the family of Meliaceae and originates from India and Myanmar. Evidence indicates that *A. indica* was used in ancient times to cure cholera, diarrhea, wounds, rheumatism, diabetes, eczema, ringworm, and scabies. Valuable compounds such as margosic acid, limonoids, azadirachtin, azadiradione, nimbin, salannin, stigmasterol, nimbiol, sugiol, α-terpinene terpinen-4-ol, 4-cymene, epoxyazadiradione, and vitamin E have been isolated from *A. indica* and identified [13].

Gedunin, a limonoid derived from *A. indica*, has been extensively investigated for its multifaceted biological activities, including anti-inflammatory, antimicrobial, and anticancer effects [14]. Specifically, gedunin has exhibited the potential to attenuate the production of pro-inflammatory cytokines, such as IL-1β and TNF-α, across various inflammatory models [15,16,17]. Previous studies have indicated that gedunin exerts its anti-inflammatory effects primarily through the inhibition of the NF-κB signaling pathway, which is crucial for the regulation of pro-inflammatory genes [15]. Furthermore, gedunin suppresses the activation of the NLRP3 inflammasome, further elucidating its capacity to mitigate inflammation at multiple signaling levels [18]. In the context of skin inflammation, research focusing on other inflammatory conditions, such as arthritis and allergic airway inflammation, has established the efficacy of gedunin in reducing cellular oxidative stress and cytokine-mediated immune responses [16,17]. These findings provide a robust rationale for investigating gedunin’s potential therapeutic role in acne-related inflammation.

We conducted a screening of natural compounds to identify inhibitors of *C. acnes*-induced inflammation in a RAW 264.7 murine macrophage cell line, leading to the identification of gedunin. The anti-inflammatory effects of gedunin and its underlying signaling transduction mechanisms were further evaluated using the RAW264.7 cell line, primary macrophages, and a murine model.

## 2. Results

### 2.1. Gedunin Suppresses C. acnes-Induced Inflammation in RAW 264.7 Macrophages

**Figure 1:**
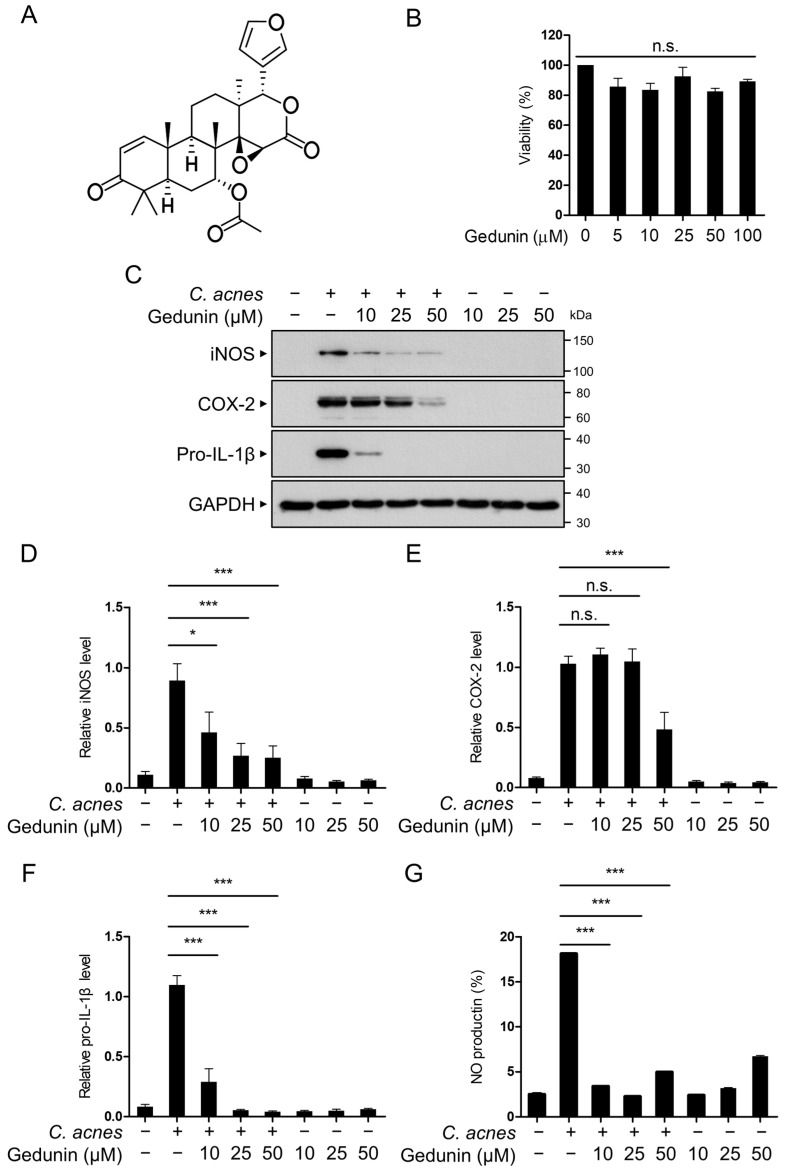
Inhibitory effects of gedunin on *C. acnes*-induced inflammatory response in vitro. (**A**) Chemical structure of gedunin. (**B**) Cell viability of RAW 264.7 cells was evaluated using the trypan blue assay after treatment with various concentrations of gedunin (0, 5, 10, 25, 50, and 100 µM) for 12 h. (**C**–**F**) RAW 264.7 cells were pretreated with gedunin (10, 25, and 50 μM) for 30 min, followed by exposure to heat-killed *C. acnes* (1 × 10^6^ CFU/mL) for 6 h. Protein expression levels of iNOS, COX-2, pro-IL-1β, and GAPDH were determined by Western blotting and quantified. (**G**) NO levels were measured using the Griess reagent. The data are expressed as the mean ± standard deviation (SD) based on three independent experiments. n.s., not significant; * *p* < 0.05, *** *p* < 0.001.

To investigate the effects of gedunin on *C. acnes*-induced inflammatory signaling pathways, we first assessed the non-cytotoxic concentration of gedunin in RAW 264.7 murine macrophage cells. The chemical structure of gedunin is shown in Figure 1A. RAW 264.7 cells were treated with gedunin at concentrations of 10, 25, and 50 μM for 12 h, and cell viability was assessed using the MTT assay. The results indicated that treatment with gedunin at a maximum concentration of 50 μM for 12 h did not affect cell viability (Figure 1B). Based on these findings, subsequent studies were conducted using gedunin concentrations of 10, 25, and 50 μM.

Next, we examined the inhibitory effects of gedunin on the protein expression of the pro-inflammatory mediators induced by *C. acnes*. The results confirmed that gedunin inhibited the expression of iNOS, COX-2, and pro-IL-1β proteins in a dose-dependent manner (Figure 1C–F). These findings indicate that gedunin has anti-inflammatory effects on the inflammatory signaling pathways triggered by *C. acnes*. Furthermore, based on the observed reduction in iNOS protein expression, we performed an NO assay using Griess reagent to verify the decrease in NO production induced by *C. acnes* (Figure 1G). Collectively, these results indicate that gedunin suppresses the expression of *C. acnes*-induced pro-inflammatory mediators.

### 2.2. Gedunin Suppresses mRNA Expression of Inflammatory Mediators Induced by C. acnes

**Figure 2:**
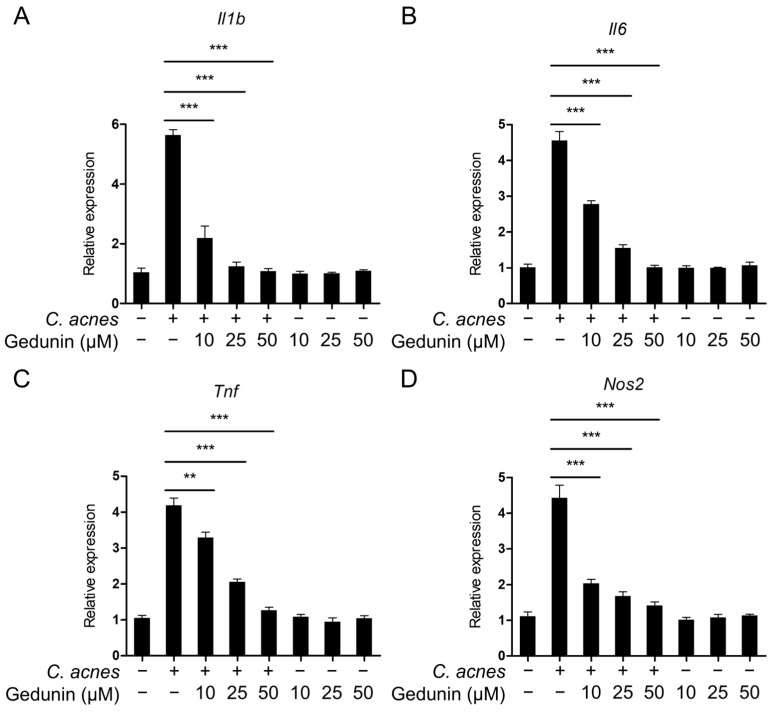
Gedunin suppresses the mRNA levels of inflammatory mediators induced by *C. acnes*. (**A**–**D**) RAW 264.7 cells were pretreated with gedunin at concentrations of 10, 25, and 50 μM for 30 min, followed by incubation with heat-killed *C. acnes* (1 × 10^6^ CFU/mL) for 6 h. The mRNA expression levels of *Il1b*, *Il6*, *Tnf*, and *Nos2* were then measured using quantitative real-time PCR. The data are expressed as the mean ± standard deviation (SD) based on three independent experiments. ** *p* < 0.01, *** *p* < 0.001.

The impact of gedunin on the mRNA expression of inflammatory mediators triggered by *C. acnes* was assessed using quantitative real-time PCR (qPCR). The analysis revealed a dose-dependent reduction in the mRNA levels of key *C. acnes*-induced inflammatory mediators, including *Il1b*, *Il6*, *Tnf*, and *Nos2*(Figure 2A–D). These results suggest that gedunin effectively inhibits *C. acnes*-induced inflammatory signaling pathways.

### 2.3. Gedunin Suppresses the NF-κB Signaling Pathway Induced by C. acnes

**Figure 3:**
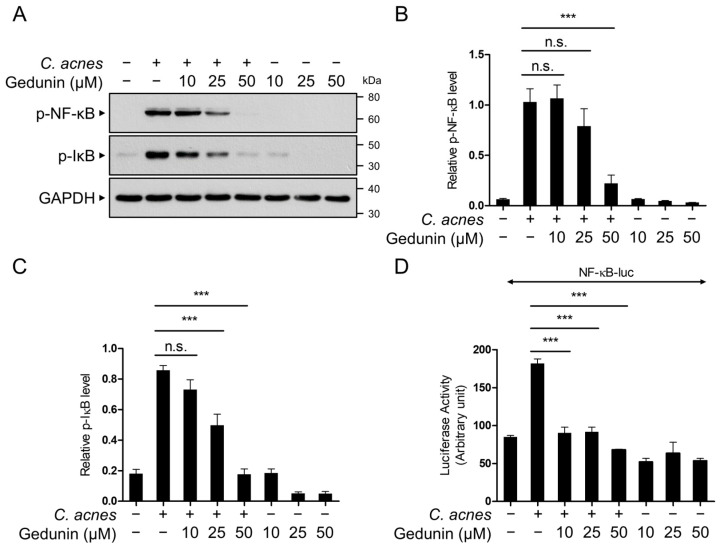
Gedunin inhibits the activation of the NF-κB signaling pathway induced by *C. acnes*. RAW 264.7 cells were pretreated with gedunin at concentrations of 10, 25, and 50 μM for 30 min, followed by incubation with heat-killed *C. acnes* (1 × 10^6^ CFU/mL) for 6 h. (**A**) Protein levels of phospho-NF-κB (p65), phospho-IκB, and GAPDH were analyzed by Western blotting, and (**B**,**C**) the relative intensities of the bands were quantified. (**D**) RAW 264.7 cells were transfected with the NF-κB-luciferase reporter plasmid (NF-κB-luc). After 24 h of transfection, the cells were pretreated with gedunin for 30 min, followed by incubation with heat-killed *C. acnes* (1 × 10^6^ CFU/mL) for 6 h. The data are expressed as the mean ± standard deviation (SD) based on three independent experiments. n.s., not significant; *** *p* < 0.001.

The NF-κB signaling pathway is a well-established regulator of inflammatory cytokine expression, including IL-1β, COX-2, and iNOS [8]. To evaluate whether gedunin inhibits the *C. acnes*-induced NF-κB signaling pathway, we analyzed the phosphorylation of NF-κB and IκB using Western blotting. The results revealed a dose-dependent inhibition of NF-κB and IκB phosphorylation by gedunin (Figure 3A–C). Additionally, a reporter gene assay confirmed that gedunin significantly reduced *C. acnes*-induced NF-κB transcriptional activity (Figure 3D). These findings indicate that the anti-inflammatory effects of gedunin are mediated through the suppression of the NF-κB signaling pathway.

### 2.4. Gedunin Does Not Affect the MAPK Signaling Pathway

**Figure 4:**
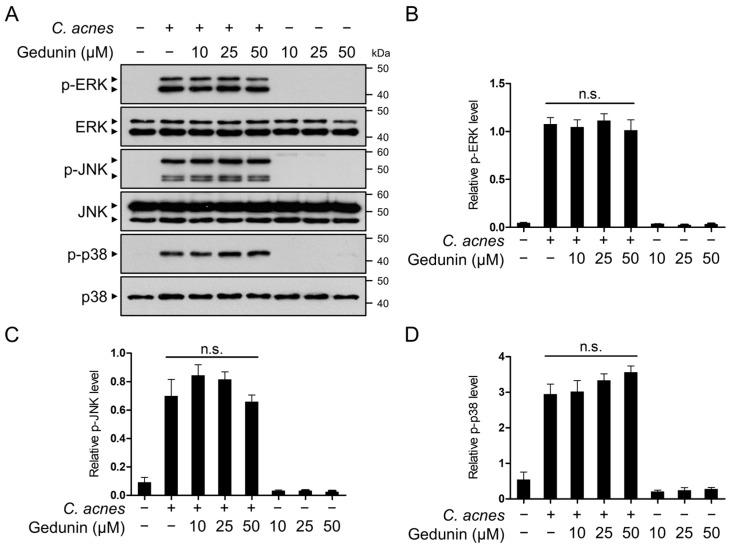
Gedunin does not inhibit the activation of MAPK signaling pathways induced by *C. acnes*. (**A**–**D**) RAW 264.7 cells were pretreated with gedunin at concentrations of 10, 25, and 50 μM for 30 min, followed by incubation with heat-killed *C. acnes* (1 × 10^6^ CFU/mL) for 6 h. (**A**) Protein levels of phospho-ERK, ERK, phospho-JNK, JNK, phospho-p38, and p38 were analyzed by Western blotting, and (**B**–**D**) the relative intensities of the bands were quantified. The data are expressed as the mean ± standard deviation (SD) based on three independent experiments. n.s., not significant.

The MAPK signaling pathway, including the activation of JNK, ERK, and p38, plays a key role in the TLR2-mediated response to *C. acnes* [8]. To investigate whether gedunin inhibits the *C. acnes*-induced MAPK signaling pathway, Western blot analysis was performed to evaluate the phosphorylation levels of ERK, JNK, and p38. The results demonstrated that gedunin had no effect on the phosphorylation of these MAPKs (Figure 4A–D). These findings suggest that the anti-inflammatory effects of gedunin are independent of the MAPK signaling pathway.

### 2.5. Gedunin Inhibits C. acnes-Induced Activation of NLRP3 Inflammasome

**Figure 5:**
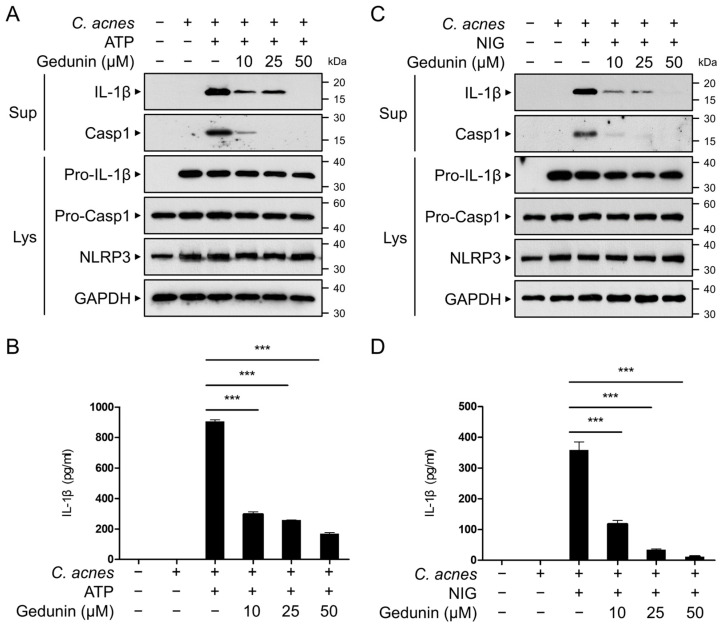
Gedunin inhibits the activation of NLRP3 inflammasome. (**A**,**C**) Mouse BMDMs were primed with heat-killed *C. acnes* (1 × 10^6^ CFU/mL) for 3 h. The cells were then pretreated with varying concentrations of gedunin for 30 min, followed by stimulation with ATP (5 mM) or NIG (10 μM) for 1 h. Culture supernatants (Sup) and cell lysates (Lys) were analyzed by immunoblotting with anti-IL-1β, anti-NLRP3, anti-caspase-1, and anti-β-actin antibodies. (**B**,**D**) Secreted IL-1β protein levels in the supernatants were measured using ELISA. The data are expressed as the mean ± standard deviation (SD) based on three independent experiments. *** *p* < 0.001.

The maturation of pro-IL-1β into its active form is mediated by inflammasomes, after which the active IL-1β is secreted from the cell. To determine whether gedunin suppresses the secretion of active IL-1β, bone-marrow-derived macrophages (BMDMs) were treated with ATP or NIG to activate the NLRP3 inflammasome. Western blot analysis of the culture supernatants revealed that gedunin reduced the levels of secreted active IL-1β and cleaved caspase-1 in a dose-dependent manner (Figure 5A,C). Furthermore, ELISA confirmed that gedunin treatment significantly decreased IL-1β secretion in the culture supernatants (Figure 5B,D). These results indicate that gedunin suppresses the activation of the NLRP3 inflammasome, contributing to its anti-inflammatory effects.

### 2.6. Gedunin Alleviates C. acnes-Induced Skin Inflammation In Vivo

**Figure 6:**
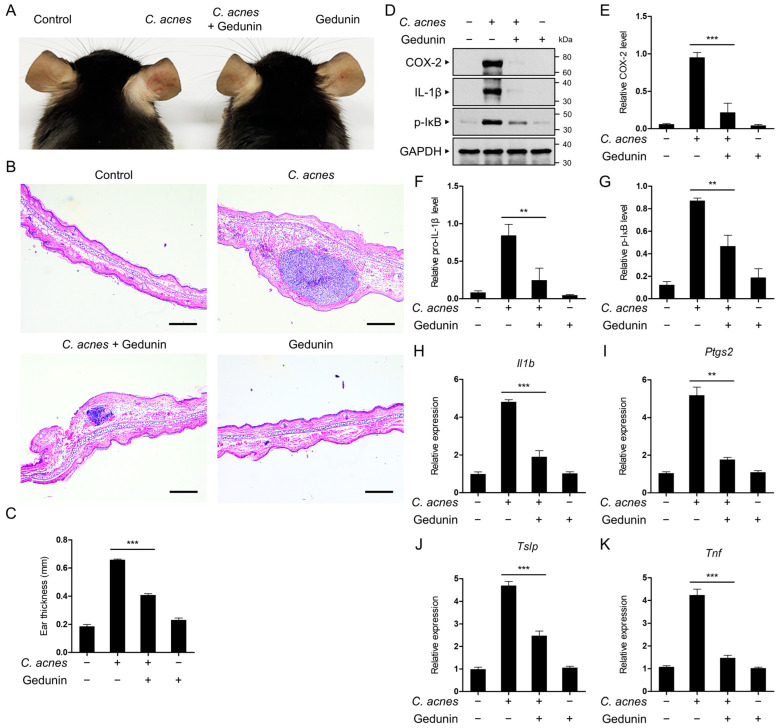
Inhibitory effects of gedunin in a mouse acne model. Mice were injected in the ear with *C. acnes* (1 × 10^8^ CFU in 20 μL PBS), with or without co-administration of gedunin (25 mg/kg). (**A**) Photographs of mouse ears taken 24 h post-injection. (**B**) Formalin-fixed ear tissues were processed, embedded, sectioned, and stained with H&E for histopathological examination. Scale bar: 200 μm. (**C**) Ear thickness was measured to assess swelling. (**D**–**G**) Ear tissues were analyzed by immunoblotting with anti-COX-2, anti-IL-1β, anti-phospho-IκB, and anti-GAPDH antibodies, and the relative protein levels were quantified. (**H**–**K**) The mRNA expression levels of *Il1b*, *Ptgs2*, *Tslp*, and *Tnf* in ear tissues were measured using qRT-PCR. The data are expressed as the mean ± standard deviation (SD) based on three independent experiments. ** *p* < 0.01, *** *p* < 0.001.

The anti-inflammatory effects of gedunin were further evaluated in vivo using a mouse acne model. Mice were injected with *C. acnes* into the ear, and histological changes were assessed 24 h later. Mice injected with *C. acnes* alone displayed typical inflammatory symptoms, including erythema, whereas the co-injection with gedunin significantly reduced erythema (Figure 6A).

Histopathological analysis using hematoxylin and eosin (H&E) staining revealed that *C. acnes* inoculation caused increased ear thickness due to swelling and the infiltration of inflammatory cells into the dermis. These effects were mitigated in mice treated with gedunin (Figure 6B,C). Western blot analysis of the ear tissues showed elevated levels of pro-IL-1β and COX-2 protein expression, as well as IκB phosphorylation, in the *C. acnes*-injected group. In contrast, these increases were markedly suppressed in the gedunin-treated group (Figure 6D–G).

Additionally, mRNA levels of pro-inflammatory mediators, including IL-1β, COX-2, thymic stromal lymphopoietin (TSLP), and TNF-α, were significantly reduced in the ear tissues of mice treated with gedunin (Figure 6H–I). These findings indicate that gedunin effectively inhibits *C. acnes*-induced skin inflammation in vivo, consistent with its anti-inflammatory effects observed in vitro.

## 3. Discussion

Isotretinoin, a derivative of vitamin A, is widely utilized for acne due to its efficacy in reducing inflammation and regulating skin cell turnover [8]. However, its use comes with significant limitations, such as teratogenic risk, which necessitates strict contraception during the treatment period. Additionally, individuals undergoing treatment are advised against blood donation due to the potential risks associated with the medication [19]. Another commonly employed approach involves antibiotics like tetracycline, doxycycline, and erythromycin, which target the bacterial component of acne [20]. Despite their effectiveness, these antibiotics can lead to severe side effects, including organ damage and hypersensitivity reactions, such as contact dermatitis. Moreover, the prolonged or improper use of antibiotics raises concerns about the emergence of antibiotic-resistant bacteria, further complicating treatment options [20,21]. To address these challenges, recent research has shifted focus toward the identification and development of natural substances with strong antimicrobial activity. These alternatives are being explored for their ability to combat acne while offering advantages such as low toxicity and minimal adverse effects, making them promising candidates for safer and more sustainable acne management strategies.

Gedunin, a naturally occurring tetranortriterpenoid predominantly found in plants of the Meliaceae family, exhibits diverse biological activities. It has been studied for its potential in treating various diseases, including cancer, neurodegenerative conditions, inflammation, diabetes, and microbial infections [14]. Gedunin exerts anticancer effects by targeting Heat Shock Protein 90 (Hsp90), a molecular chaperone that stabilizes multiple oncogenic proteins [22]. This inhibition leads to apoptosis and halts cancer cell proliferation in ovarian, breast, prostate, and pancreatic cancers. Its ability to counteract chemoresistance further highlights its therapeutic promise [23]. Gedunin shows neuroprotective potential in neurodegenerative conditions like Alzheimer’s and Parkinson’s diseases [15]. It reduces neuroinflammation and oxidative stress by activating Nrf2 signaling and inhibiting pro-inflammatory pathways, which makes it a promising candidate for neurodegenerative disease therapies. By inhibiting pancreatic α-amylase, gedunin reduces glucose release from dietary starch and enhances insulin sensitivity, suggesting its utility in managing diabetes [24]. Gedunin exhibits antimicrobial properties against pathogens like *Xylella fastidiosa* and antiparasitic effects, particularly against *Plasmodium falciparum* [25,26]. Previous studies suggest its efficacy in reducing symptoms in diverse inflammatory models, such as arthritis and allergic airway inflammation, confirming its role as an anti-inflammatory agent [16,17].

**Figure 7:**
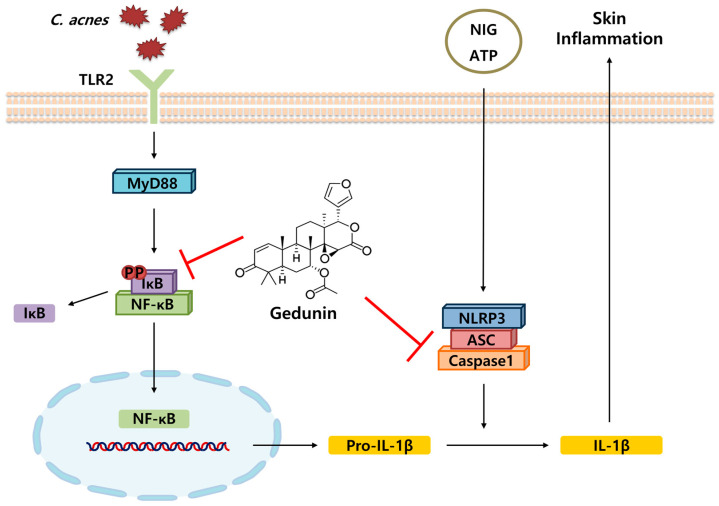
Schematic representation of the mechanism underlying the gedunin-mediated inhibition of *C. acnes*-associated inflammatory signaling pathways. *C. acnes* stimulates TLR2, leading to the recruitment of MyD88 and the activation of NF-κB. Activated NF-κB translocates to the nucleus, promoting the transcription of pro-IL-1β. In the presence of signals such as ATP or Nigericin (NIG), the NLRP3 inflammasome is activated, resulting in the cleavage of pro-IL-1β to mature IL-1β by caspase-1. IL-1β then contributes to skin inflammation. The red symbols in the figure represent inhibition by Gedunin, which blocks both the activation of NF-κB and NLRP3 inflammasome assembly, thereby suppressing the production of IL-1β and preventing inflammation.

Along with these reports, our study presents novel evidence demonstrating that gedunin effectively suppresses the skin inflammatory response induced by acne-causing bacteria. Gedunin inhibited the expression of key inflammatory mediators, including nitric oxide, IL-1β, and TNF-α, in both in vitro and in vivo experimental models by suppressing the TLR2-mediated activation of the NF-kB signaling pathway (Figure 7). The findings of this study revealed that gedunin exerts significant anti-inflammatory effects on *C. acnes*-induced skin inflammation by targeting the NF-κB signaling pathway and the NLRP3 inflammasome. This is consistent with existing research highlighting the broad biological activities of gedunin, including its anticancer, anti-inflammatory, and antimicrobial properties. However, our study offers a novel perspective by demonstrating the efficacy of gedunin in acne-related skin inflammation, an area that has not been extensively explored in the previous literature. Compared to other natural anti-inflammatory compounds, gedunin’s selective inhibition of the NF-κB and NLRP3 inflammasome pathways offers a distinct advantage. For example, curcumin, a well-known natural anti-inflammatory agent, targets multiple pathways, including NF-κB, MAPK, and JAK/STAT, but often requires higher doses to achieve similar efficacy [27,28]. Likewise, resveratrol exhibits strong anti-inflammatory effects but is limited by its low bioavailability [29,30]. In contrast, gedunin demonstrated significant effects at relatively low concentrations in our study, highlighting its therapeutic potential.

Preclinical evidence suggests that gedunin has low toxicity to normal cells, making it relatively safe. However, more extensive studies, including preclinical and clinical trials, are necessary to confirm its safety and efficacy in humans. Gedunin’s broad biological activities make it a valuable compound for therapeutic development. However, challenges such as limited availability, poor bioavailability, and production difficulties need to be addressed. Future studies should explore synthetic or semi-synthetic derivatives, improve its pharmacokinetic properties, and investigate combination therapies for enhanced effectiveness. Moreover, the impact of gedunin on the skin microbiome warrants further investigation. Since *C. acnes* is an integral component of the skin microbiota, understanding how gedunin modulates microbial communities could offer valuable insights into its long-term effects and safety.

## 4. Materials and Methods

### 4.1. Materials

Gedunin (molecular formula: C_28_H_34_O_7_; purity > 95%; Chemical Abstracts Service (CAS) number: 2753-30-2) was purchased from Biosynth (Staad, Switzerland). ATP was purchased from Sigma-Aldrich (St. Louis, MO, USA). Nigericin (NIG) was purchased from Tocris (Bristol, UK). Anti-phospho-JNK (#4668), anti-JNK (#9252), anti-phospho-p38 (#4511), anti-p38 (#9212), anti-phospho-ERK (#9106), anti-COX2 (#12282), anti-iNOS (#13120), anti-phospho-NF-κB (#3033), and anti-phospho-IκB (#9246) antibodies were purchased from Cell Signaling Technology (Danvers, MA, USA). Anti-ERK (sc-153) and anti-GAPDH (sc-32233) antibodies were purchased from Santa Cruz Biotechnology (Dallas, TX, USA). Anti-caspase-1 (AG-20B-0042) and anti-NLRP3 (AG-20B-0014) antibodies were purchased from Adipogen Life Sciences (San Diego, CA, USA). Anti-IL-1β (AF-401-NA) antibody was purchased from R&D Systems (Minneapolis, MN, USA).

### 4.2. C. acnes

*C. acnes* (KCTC3314) was obtained from the Korean Culture Center of Microorganisms (Seoul, Republic of Korea) and grown in reinforced clostridial medium (Merck Millipore, Darmstadt, Germany). The bacteria were cultivated anaerobically at 37 °C using an anaerobic Gas-Pak system. After cultivation, *C. acnes* cells were harvested by centrifugation (VS-550, VISION Scientific Co., Daejeon, Republic of Korea) at 3000× *g* for 20 min at 4 °C, and the resulting bacterial pellets were washed with PBS prior to further use.

### 4.3. Cell Culture

RAW 264.7 macrophages were obtained from the American Type Culture Collection (ATCC, Rockville, MD, USA) and maintained in DMEM containing 1% penicillin and streptomycin and 10% heat-inactivated fetal bovine serum (FBS, all from Thermo Fisher Scientific, San Jose, CA, USA). BMDMs were prepared according to the method described in our previous work [31].

### 4.4. Cell Viability Assay

RAW 264.7 cells were plated on 12-well plates. After 24 h, the cells were treated with gedunin at the indicated concentrations and incubated at 37 °C for 12 h. To determine the number of live cells per well, the cells were stained with trypan blue dye (Sigma-Aldrich, St. Louis, MO, USA) and counted using a hemocytometer.

### 4.5. Western Blot Analysis

RAW 264.7 cells were seeded into 12-well plates and maintained at 37 °C with 5% CO_2_ for 24 h to allow stabilization. The cells were pretreated with gedunin before being exposed to heat-killed *C. acnes* (1 × 10^6^ CFU/mL). To assess NLRP3 inflammasome activation, the cells were treated with either 5 mM ATP or 10 μM NIG for 1 h. The culture supernatants were collected into tubes and centrifuged at 2000× *g* for 5 min to remove debris. The remaining cells were lysed in a buffer containing 10% glycerol, protease inhibitors, 10 mM NaF, 1% Nonidet P-40, 150 mM NaCl, 50 mM Tris-Cl (pH 8.0), 1 mM Na_3_VO_4_, 1 mM EGTA, and 0.2 mM PMSF. The lysates were incubated on ice for 30 min before being centrifuged, and the resulting supernatant was collected for protein analysis. Proteins were separated using SDS-PAGE (Bio-Rad, Hercules, CA, USA) and transferred onto a polyvinylidene fluoride (PVDF) membrane (Cytiva, Marlborough, MA, USA). After blocking with TBS-T (LPS solution, Daejeon, Republic of Korea) containing 5% skim milk, the membranes were incubated with the primary antibody overnight at 4 °C, followed by a 1 h incubation with the appropriate secondary antibody. Protein bands were detected using an enhanced chemiluminescence reagent (Thermo Fisher Scientific, San Jose, CA, USA).

### 4.6. NO Assay

To measure the NO production induced by *C. acnes*, RAW 264.7 cells were cultured on a plate and stabilized at 37 °C in a 5% CO_2_ incubator for 24 h. The cells were then pretreated with gedunin for 30 min, followed by incubation with *C. acnes* for 6 h. After 24 h, 50 μL of the culture supernatant was transferred to a 96-well plate and mixed with 50 μL of Griess reagent (Sigma-Aldrich, St. Louis, MO, USA). After allowing the reaction to proceed for 10 min, absorbance was measured at a wavelength of 540 nm using a SpectraMax iD5 multi-mode microplate reader (Molecular Devices, Sunnyvale, CA, USA).

### 4.7. Quantitative Real-Time PCR

Total RNA was isolated using TRIzol reagent (Invitrogen, Carlsbad, CA, USA), and complementary DNA (cDNA) was synthesized with the ReverTra Ace qPCR RT Master Mix containing gDNA Remover (TOYOBO, Osaka, Japan). The cDNA was subsequently amplified using SYBR Green Real-Time PCR Master Mix (TOYOBO). The primer sequences used for amplification are provided in Table 1. Each sample was analyzed in triplicate, and *β-actin* was used as the reference gene for normalizing mRNA expression levels. The AriaMX system (Agilent, Santa Clara, CA, USA) was employed for data analysis.

### 4.8. Reporter Gene Assay

RAW 264.7 cells were transfected with NF-κB-Luc and *Renilla* reporter plasmids using Lipofectamine 3000 (Invitrogen, Waltham, MA, USA). Following a 24 h incubation period, the cells were treated with gedunin before being exposed to heat-killed *C. acnes* (1 × 10^6^ CFU/mL). Luciferase activity, reflecting transcriptional activation, was measured using the Dual-Luciferase Assay System (Promega, Madison, WI, USA), a well-established method for evaluating gene reporter assays.

### 4.9. ELISA

BMDM culture supernatants were collected and centrifuged at 2000× *g* for 10 min at 4 °C, and the concentration of IL-1β was measured according to the manufacturer’s instructions (BioLegend, San Diego, CA, USA).

### 4.10. In Vivo Mouse Acne Model

C57BL/6 mice, obtained from Orient Bio Inc. (Seongnam, Republic of Korea), were housed in a controlled environment at the Animal Center of Kangwon National University. All procedures were conducted with the approval of the Institutional Animal Care and Use Committee (IACUC, KW-231127-2, Kangwon National University, Republic of Korea). Groups of 7 mice each were administered ear injections with one of the following: PBS alone, live *C. acnes* (1 × 10^8^ CFU in 20 μL PBS), live *C. acnes* combined with gedunin (25 mg/kg), or gedunin alone. After 24 h, the mice were euthanized, and ear tissues were harvested for subsequent analysis.

### 4.11. Histological Analysis

For histological analysis, ear tissue samples were fixed in 4% formaldehyde and embedded in paraffin. Paraffin blocks were sectioned at a thickness of 2–3 μm and stained with hematoxylin and eosin (H&E staining kit, ab245880, Abcam, Cambridge, MA, USA). Photomicrographs were obtained using an Olympus microscope (Tokyo, Japan).

### 4.12. Statistics

Western blot densitometry was performed and quantified using ImageJ software version 1.52a (NIH, Bethesda, MD, USA). Data analysis was conducted using GraphPad Prism 5.01 (GraphPad Software, Inc., San Diego, CA, USA), and the results are presented as the mean ± standard deviation (SD). Statistical comparisons between the experimental and control groups were made using Student’s *t*-test, while differences among multiple groups were assessed through ANOVA, followed by Bonferroni post hoc tests. A *p*-value of less than 0.05 was considered statistically significant, with significance levels indicated as follows: * *p* < 0.05, ** *p* < 0.01, and *** *p* < 0.001.

## 5. Conclusions

Our findings reveal that gedunin effectively mitigates inflammation by suppressing NF-κB activation in both in vitro and in vivo models. Furthermore, it inhibits IL-1β maturation through the NLRP3 inflammasome, highlighting its broad anti-inflammatory properties. This study provides the first scientific evidence that gedunin can attenuate skin inflammation caused by *C. acnes*, underscoring its potential as a promising therapeutic agent for acne treatment.

## Figures and Tables

**Table 1:** List of primers used for qRT-PCR.

Gene	Primer Sequence	Accession No.	Product Size (bp)
*Il1b* (IL-1β)	F: GCCACCTTTTGACAGTGATGAG	NM_008361.4	165
	R: AGTGATACTGCCTGCCTGAAG		
*Il6* (IL-6)	F: TACCACTTCACAAGTCGGAGGC	NM_031168.2	116
	R: CTGCAAGTGCATCATCGTTGTTC		
*Tnf* (TNF-α)	F: CCCTCACACTCACAAACCAC	NM_001278601.1	133
	R: ACAAGGTACAACCCATCGGC		
*Nos2* (iNOS)	F: TCCTGGACATTACGACCCCT	NM_001313922.1	148
	R: AGGCCTCCAATCTCTGCCTA		
*Ptgs2* (Cox-2)	F: TTGGAGGCGAAGTGGGTTTT	NM_011198.5	148
	R: TGGGAGGCACTTGCATTGAT		
*Tslp* (TSLP)	F: CCCTTCACTCCCCGACAAAA	NM_021367.2	61
	R: GCAGTGGTCATTGAGGGCTT		
*Actb* (β-actin)	F: AGAGGGAAATCGTGCGTGAC	NM_007393.5	138
	R: CGATAGTGATGACCTGACCGT		

## Data Availability

The original contributions presented in the study are included in the article, further inquiries can be directed to the corresponding authors.
